# Host Non-Coding RNA Regulates Influenza A Virus Replication

**DOI:** 10.3390/v14010051

**Published:** 2021-12-29

**Authors:** Yuejiao Liao, Shouqing Guo, Geng Liu, Zhenyu Qiu, Jiamin Wang, Di Yang, Xiaojing Tian, Ziling Qiao, Zhongren Ma, Zhenbin Liu

**Affiliations:** 1 Gansu Tech Innovation Center of Animal Cell, Biomedical Research Center, Northwest Minzu University, Lanzhou 730030, China; lyj1997052@163.com (Y.L.); guoshouqing292196@163.com (S.G.); lg13619271425@163.com (G.L.); Qiuzhenyu9810@163.com (Z.Q.); jiaminwang1987@163.com (J.W.); xbmzyd@163.com (D.Y.); qiaozilin@xbmu.edu.cn (Z.Q.); mzr@xbmu.edu.cn (Z.M.); 2 Life Science and Engineering College, Northwest Minzu University, Lanzhou 730030, China; smile_tian@yeah.net; 3 Key Laboratory of Biotechnology & Bioengineering of State Ethnic Affairs Commission, Biomedical Research Center, Northwest Minzu University, Lanzhou 730030, China

**Keywords:** IAV, miRNA, lncRNA, circRNA, interferon, antiviral innate immune response

## Abstract

Outbreaks of influenza, caused by the influenza A virus (IAV), occur almost every year in various regions worldwide, seriously endangering human health. Studies have shown that host non-coding RNA is an important regulator of host–virus interactions in the process of IAV infection. In this paper, we comprehensively analyzed the research progress on host non-coding RNAs with regard to the regulation of IAV replication. According to the regulation mode of host non-coding RNAs, the signal pathways involved, and the specific target genes, we found that a large number of host non-coding RNAs directly targeted the PB1 and PB2 proteins of IAV. Nonstructural protein 1 and other key genes regulate the replication of IAV and indirectly participate in the regulation of the retinoic acid-induced gene I-like receptor signaling pathway, toll-like receptor signaling pathway, Janus kinase signal transducer and activator of transcription signaling pathway, and other major intracellular viral response signaling pathways to regulate the replication of IAV. Based on the above findings, we mapped the regulatory network of host non-coding RNAs in the innate immune response to the influenza virus. These findings will provide a more comprehensive understanding of the function and mechanism of host non-coding RNAs in the cellular anti-virus response as well as clues to the mechanism of cell–virus interactions and the discovery of antiviral drug targets.

## 1. Introduction

Influenza viruses belong to the Orthomyxoviridae RNA virus family and is divided into genera A, B, C and D. Among them, genera A, B and C cause respiratory diseases in humans [1]. Influenza A virus (IAV) can infect a wide range of hosts and is highly infectious & transmissible, and easily mutable, which can result in an influenza pandemic in a short time; therefore, it creates an enormous burden and pressure on the public health system [2,3]. The innate immune response is the first line of defense against virus infection and plays an important role in the process of resisting virus invasion [4]. In the process of IAV infection, the body relies on different pattern recognition receptors (PRRs) in cells to recognize virus-related molecular patterns, such as retinoic acid-induced gene I (RIG-I), melanoma differentiation factor 5 (MDA5), and toll-like receptors (TLRs). Many different molecular signals (such as: RIG-I, MDA5, TRAF3, IKK, TBK1) are also recruited to inhibit the replication of the influenza virus through a series of complex signal pathways, including PRR-dependent signal pathways, which can induce the production of interferon (IFN) and inflammatory factors, as well as the expression of antiviral IFN-stimulated genes (ISGs) [5,6,7,8]. These proteins not only inhibit the replication of viruses in infected cells but also recruit dendritic cells (DCs) and macrophages from virus-infected tissues and further stimulate the immune response mediated by T cells and B cells [9,10].

**Figure 1:**
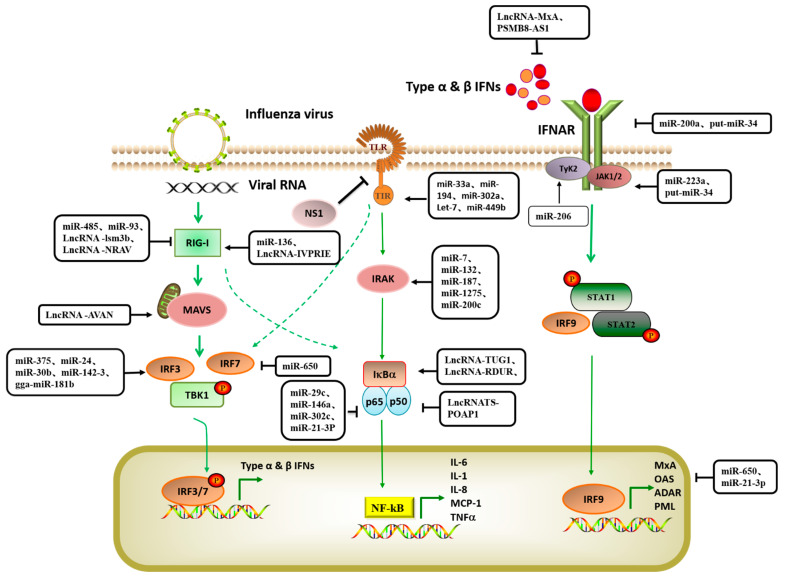
Host noncoding RNA involved in regulating influenza A virus response signal pathway. The natural immune response is the body’s first line of defense against viral infection and involves the activation of multiple antiviral signal pathways as well as the activation and expression of antiviral factors. During this process, host noncoding RNAs are induced or inhibited by influenza virus infection, which mediates the activation of target genes, affects the expression of antiviral natural immune molecules, and indirectly regulates the replication and proliferation of influenza virus. The target gene sites in the natural immune response signaling pathway involved in the regulation of noncoding RNA are concentrated in the RLR signaling pathway, TLR-like receptor signaling pathway, JAK-STAT signal pathway, and NF-κB signaling pathway. (Dotted arrow is indirect effect; solid line is direct effect).

Non-coding RNAs (ncRNAs) are a large class of RNA transcripts that are transcribed from the genome but lack the ability to encode proteins [11]. At the RNA level, they can perform their respective biological functions and play an important role in cell growth, differentiation, replication, and apoptosis. Regulatory non-coding RNAs mainly include microRNA (miRNA), long non-coding RNA (lncRNA)and circular RNA (circRNA) [12,13]. MiRNA, lncRNA, and other non-coding RNA participate in gene expression regulation, thereby affecting cell replication and differentiation, individual growth and development, immune response, and other life activities [14,15,16]. Recent studies have shown that non-coding RNAs play an important role in regulating the interactions between host cells and viruses [17,18,19]. Influenza virus infection can induce significant changes in the expression of many hosts’ non-coding RNAs, some of which are involved in regulating the antiviral response of host cells, while others promote the efficient replication of the virus [20,21]. In this paper, we systematically summarized the host non-coding RNAs that are involved in the regulation of influenza virus replication, performed summary analysis according to their inhibition or promotion of virus replication, and mapped the signal network of host non-coding RNAs that regulate the influenza virus response (see Figure 1 for relevant information).

## 2. Host miRNA Regulates IAV Replication

MiRNAs are widely distributed in eukaryotes and some viral genomes. They are highly conserved non-coding single-stranded RNA fragments encoded by nuclear genes and play a role in the regulation of post-transcriptional gene expression [22,23]. In animal cells, miRNA mediates the inhibition of target gene expression or mRNA degradation by recognizing the 5′-UTR—3′-UTR of target gene mRNA to form a 2–8nt complementary structure and also regulates the expression of key genes in different signaling pathways [24,25]. Analyzing the miRNA reported to be involved in the regulation of IAV revealed that the host miRNA regulation of viral gene expression can be divided into two parts. First, the miRNA directly targets the mRNAs of the influenza virus, inhibits the expression of viral proteins, or mediates the degradation of viral mRNAs, thereby inhibiting the replication of the virus. Second, the miRNA indirectly regulates the replication of the influenza virus by targeting key genes in the influenza virus response signaling pathway.

### 2.1. Host miRNA Directly Inhibits IAV Gene Expression

From the perspective of IAV infection, cell miRNA expression is crucial for virus–host interactions such as virus entry, replication, translation, and transmission. When H9N2 or H5N1 infected avian-derived DCs, gga-miR6675 and gga-miR6616 were found to target of mRNA nuclear localization sequence (NLS) of viral PB1, triggering PB1 gene silencing and inhibiting H9N2 replication. (See Table 1 for relevant information) The study may provide new insights into the interactions between bird miRNA and bird DCs and the inhibition of virus replication [26]. In A549 cells with high expression of miR-3145, miR-3145 can silence the 3′-UTR of the PB1 gene for the H5N1, H1N1, and H3N2 subtypes of IAV, reduce gene expression and inhibit virus replication [27]. In addition, miR-323, miR-491, and miR-654 contain the same nucleotide sequence, which can down-regulate the expression of the PB1 gene through the conserved region of the PB1 gene for the H1N1 virus, thereby inhibiting the replication of the virus in Madin-Darby canine kidney cells [28]. In A549 cells, miR-188-3p inhibited the gene expression of PB2 by binding to its mRNA and effectively inhibited the replication of IAV (H1N1, H5N6, and H7N9) [29]. Host non-coding RNAs, such as miR-584-5p [30] and miR-485 [31] have been reported to act on the PA, PB1, and PB2 genes of IAV, inhibiting mRNA expression thereby reducing virus replication.

In addition to regulating the expression of the PA, PB1, and PB2 genes of IAV, host miRNA can also inhibit the replication of IAV by binding to different target gene sites of the influenza virus, thus reducing the risk of influenza virus escape. NS1 protein of influenza A virus has been shown to induce cell cycle stagnation in the G0/G1 phase, providing a more favorable cell environment for virus replication [32]. It has been reported that hsa-miR-1307-3p is a novel and effective inhibitor of NS1 expression and influenza virus replication. Type I IFN induces the up-regulation of hsa-miR-1307-3p expression during H1N1 infection of A549 cells, and hsa-miR-1307-3p directly targets the viral gene NS1. Inhibition of NS1 gene expression, can reduce the impact of virus infection on the cell cycle, and ultimately inhibit the replication of IAV [33]. Studies have shown that not only does influenza virus infection induce some miRNAs in the host to exert antiviral effects but non-host encoded miRNAs also have anti-influenza effects. miR-2911 is an atypical miRNA encoded by Honeysuckle and can negatively regulate PB2 and NS1 protein gene expression by a variety of avian influenza viruses. In mouse model tests, miR-2911 inhibited the replication of H1N1, H5N1, and H7N9 viruses in mice, prevented weight loss and death caused by an influenza virus infection and was found to be a potential and effective broad-spectrum antiviral factor against viral infection [34]. The replication and transcription of IAV depend on nucleoprotein (NP) genes [35]. The 5′ seed regions of cfa-miR-125b and cfa-miR-151 are partially complementary to the mRNA of NP and NS1 of the avian influenza H3N2 virus [36]. MiR-769-3p targets the expression of influenza virus NP protein [29], while let-7c significantly inhibits the replication of influenza virus in A549 cells by binding to the 3′-UTR region of the M gene of IAV [37]. SSC-miR-221-3p acts as a host barrier to regulate nuclear factor kappa B (NF-κB) P65 phosphorylation, inhibit the expression of anti-apoptotic protein HMBOX1, and induce apoptosis during the cross-species infection of IAV, or by targeting and silencing the hemagglutinin (HA) gene of the H5 subtype, restricting the infection and replication of influenza viruses [38].

**Table 1:** miRNA directly targets key genes of viruses.

miRNA	Target ^a^	Cell Type	Virus	Induced Expression ^b^	Binding Site of Target ^c^	References
miR-323 miR-491 miR-654	PB1	MDCK	H1N1 (A/WSN/33)	UP	CCACC	[28]
miR-3145		A549	H5N1 (A/Thailand/NK165/2005) H1N1 (A/Thailand/104/2009) H3N2 (A/Thailand/ CU-H1817/2010)	UP	UAUGGAGCUGCCC GCUUUGGAGUGUC UUUGGAGUGUCU	[27]
miR-485		HEK293T	H5N1 (A/duck/India/02CA10/2011)	UP	CAGCCUC	[31]
miR-324-5p		A549	H5N1 (A/duck/India/02CA10/2011)	DOWN	GAGGGGAT	[39]
gga-miR6675		HEK293T	H9N2 (A/NJO2/2009)	UP	—	[26]
miR-4753		A549	H5N1 (A/Thailand/NK165/2005)	UP	AGAGAAAGAGAA	[27]
miR-584-5p	PB2	A549	H1N1 (A/Beijing/501/2009) H5N1 (A/goose/Jilin/hb/2003)	DOWN	GCAAACCA GGAGGGC	[30]
miR-1249						
miR-188-3p		A549	H1N1(A/FM/1/47) H5N6 (A/chicken/Hubei/XY918/2016) H7N9 (A/quail/Hebei/CH06–07/2018)	UP	TGTGGGA ATGTGGGA ATGTGGGA	[29]
hsa-miR-1307-3p	NS1	A549	H1N1 (A/California/04/2009)	UP	CGCCGAG	[33]
miR-3682		A549	H1N1 (A/Thailand/104/2009) H3N2 (A/Thailand/ CU-H1817/2010)	UP	GUAUCGUC AUGAUAACACAG	[27]
miR-4331		NPTR	H1N1	DOWN	TGGCCT ACAGCCAC	[40]
miR-584-5P	M1	A549	H5N1 (A/goose/Jilin/hb/2003)	UP	GCAAACCA	[30]
let-7c		A549	H1N1 (Jing Fang/86-1)	UP	ACTACCT	[37]
miR-204	HA	NPTR	H1N1 (A/swine/Nanchang/F9/2010)	DOWN	AAAGGGA	[40]
miR-192		A549	H5N1 (A/PR8)	UP	— ^d^	[41]
ssc-miR-221-3p		PAM	H5N1 (A/duck/An-hui/1/2006)	DOWN	AAUGUGGUA	[38]
miR-1249	NA	A549	H5N1 (A/goose/Jilin/hb/2003) H1N1 (A/Beijing/501/2009)	UP	GGAGGGC	[30]
miR-216b		A549	H5N1 (A/Thailand/NK165/2005) H3N2 (A/Thailand/ CU-H1817/2010)	UP	UGCAGGGAU UGUCUGCAGAGA	[27]
miR-4513	PA	A549	H1N1 (A/Thailand/104/2009) H3N2 (A/Thailand/ CU-H1817/2010)	UP	UCGUCAGUC	[27]
miR-5693		A549	H5N1 (A/Thailand/NK165 /2005) H3N2 (A/Thailand /CU-H1817/2010)	UP	UAGAGCCACUG AGAGAAAGAGAA	[27]
ssc-miR-222		HEK293T	H5N1 (A/duck/An-hui/1/2006)	UP	GAUGUGGUA	[38]

^a^: miRNA directly target key genes of viruses; ^b^: induced expression of miRNA after influenza virus infection; ^c^: sequences of miRNA binding site of target (5′-3′), ^d^: ‘—’ means unknown.

### 2.2. Host miRNAs Indirectly Inhibit IAV Replication by Regulating Intracellular Signaling

In the process of IAV infection, the body relies on different PRRs in the cell to recognize the molecular patterns associated with the virus, activate the expression of IFNs, activate the downstream signaling pathway, release a large number of inflammatory factors such as interleukins (ILs), and initiate the antiviral immune response. Studies have shown that host miRNAs can be involved in regulating the activation of host PRR-mediated antiviral signaling pathways, thereby indirectly inhibiting the replication of influenza viruses. (See Table 2 for relevant information).

Host miRNA inhibits IAV replication by regulating TLR-mediated innate immune response signaling pathways. In THP-1 cells infected with H7N9, let-7e enhanced the immune response of host cells, reduced the expression of HA, and inhibited the replication of the virus by participating in the inflammatory response mediated by the TLR4 of host cells. It also regulated the inflammatory response and mediated the anti-inflammatory process by targeting IL-1 and IL-6, preventing an excessive inflammatory response [42]. In addition, host miRNA such as miR-200a [43], miR-29c [44], miR-650 [45], miR-206 [46], and hsa-miR-664a-3p [47] have been reported to induce IFN expression by mediating TLR signaling pathways during the process of IAV infection, inhibiting the replication and proliferation of IAV. According to studies, miRNA-155 participates in the regulation of a variety of biological processes and also plays an important regulatory role in the infection of influenza virus and other viruses, mainly affecting the JAKs-STATs and TLRs/NF-κB signaling pathway to regulate viral replication and the antiviral response of the body [48,49,50]. For example, human lung microvascular endothelial cells are infected with H1N1 influenza virus, miR-155 is induced by H1N1 infection. Overexpression of miR-155 enhances the expression of inflammatory factors and activation of NF-κB factor. Sphingosine-1-phosphate receptor 1 (S1PR1) is a target of miR-155. S1PR1 is widely expressed in endothelial cells, immune cells, lymphocytes, macrophages, muscle, and other tissue cells. Mir-155 actively regulates influenza A-induced inflammation by targeting S1PR1 [51]. Another new study showed that miRNA-155-5P was effective in alleviating acute respiratory distress syndrome (ARDS) caused by influenza virus infection [52]. In addition, evidence shows that miR-155 also plays a role in the adaptive immune response induced by the influenza virus, controlling CD8+T cell response by regulating interferon signaling. The miR-155-KO dendritic cells cannot effectively present antigen and miR-155 in CD8+ T cells can control the differentiation of Th1, Th2, and Th17 subsets and affect the development of TREG cells [53].

Host miRNA inhibits IAV replication by regulating RIG-I-like receptor (RLR)-mediated innate immune response signaling pathways. RLRs play a critical role in host innate immunity, and mice with either RIG-I or MDA5 deficiency have shown susceptibility to RNA viruses [54]. When an IAV infects a host cell, viral RNA is recognized by RIG-I and MDA5, respectively, activating the signaling pathways of the innate immune response. Overexpressed RIG-I and MDA5 strongly activate the IFN-β promoter and up-regulate the expression of antiviral molecules such as 2′-5′oligoadenylate synthetase (OAS), double-stranded RNA-dependent protein kinase (PKR) and myxovirus resistance protein A (MxA) and inflammatory factors (such as IL-2, IL-6, IFN-α, and IFN-γ) [55]. MiR-136 was identified as a novel endogenous RIG-I activator that may contribute to the control of influenza virus disease. The expression of miR136 was upregulated five-fold following H5N1 influenza virus infection in A549 cells. In vitro experiments showed that miR136 exhibited strong antiviral activity against both the H5N1 influenza virus and Indiana Vesicular Stomatitis Virus (VSV). Further UTR reporter gene analysis revealed that the 3′-UTR of IL-6 is the target of miR-136, which also acts as an immune agonist for RIG-I, causing the accumulation of IL-6 and IFN-β in A549 cells. These results indicate that miR136 has dual functions of mediating post-transcriptional regulation and immune activation, enhancing the antiviral effect by promoting the expression of antiviral and inflammatory factors [56].

Host miRNA induces interferon expression by regulating the Janus kinase signal transducer and activator of transcription (JAK-STAT) signaling pathway and inhibits the replication and proliferation of IAV. The antiviral effect of IFN mainly occurs through the activation of the JAK/STAT signal, which induces the production of ISGs. IFN regulatory factor 5 (IRF-5) is a key transcription factor for maintaining the inflammatory phenotype of macrophages. IRF-5 can promote the replication of IAV, while miR-302a regulates the cytokine storm induced by IAV by binding to the 3′-UTR of IRF-5 and interferes with the replication of influenza virus [57,58]. IAVs inhibit the host type I IFN-mediated antiviral immune response by reducing the expression of miR-30, which targets and reduces the expression of suppressor of cytokine signaling 1 (SOCS1) and suppressor of cytokine signaling 3 (SOCS3), thereby reducing their inhibitory effects on the IFN/JAK/STAT signaling pathway. In addition, miR-30 inhibits the expression of the interferon-induced transmembrane proteins 3 (IFITM3) negative regulator neuronal precursor cell-expressed developmentally downregulated 4 (NEDD4) [59]. A recent study found that overexpression of miR-206 in A549 cells significantly inhibited mRNA expression of NP, NS1, and PB1 by the H1N1 and H3N2 influenza viruses, decreased the protein expression of NP and NS1, and significantly reduced influenza virus titers. The 3′-UTR reporter gene assay showed that miR-206 binding to the 3′-UTR of tankyrases2 (TNKS2) activated JNK/c-Jun signaling, induced type I IFN expression, enhanced STAT signaling, reduced the viral load in the lungs, and improved the survival rate of mice [46].

Host miRNA inhibits IAV replication through other antiviral pathways. The antigen presentation ability of DCs plays an irreplaceable role in the recognition and removal of viruses. Host miRNA can inhibit the replication of IAV by regulating the antigen presentation of host DCs. Both active and inactive H9N2 avian influenza viruses can enhance the ability of DCs to present antigens and activate T lymphocytes. GGA-miR1644 enhances the ability of DCs to inhibit viral replication by muscle blind-like protein 2. This study provides new clues for the role of miRNAs in inducing DC antigen presentation and inhibiting virus replication [26].

Host miRNA can inhibit the replication of IAV by regulating cell apoptosis. In the process of cross-species infection by the influenza virus, ssc-miR-221-3p and ssc-miR-222 can induce the apoptosis of positive allosteric modulators (PAM) cells by directly targeting the mRNA of HA and PA genes and the expression of the anti-apoptotic protein HMBOX1. Moreover, these two miRNAs inhibited the infection and replication of IAV in newborn pig trachea (NPTR) cells. Compared to Simian immunodeficiency virus (SIV) infection, P65 can be phosphorylated more efficiently after IAV infection, which is why the expression of ssc-miR-221-3p and ssc-miR-222 is significantly upregulated after viral infection [38]. According to a previous study, the stromal interaction molecule 1 (STIM1)/miR-223/nod-like receptor pyrins-3 (NLRP3) axis can regulate the inflammatory damage of lung epithelial cells induced by IAV. By inhibiting the activation of the TLR4/NF-κB signaling pathway and NLRP3 inflammasome, miR-223 alleviated the oxidative stress and apoptosis of lung epithelial cells induced by IAV and alleviated cell damage. In the serum of patients infected with IAV, STIM1 was significantly upregulated, while miR-223 was down-regulated. STIM1 regulates the expression of NLRP3 by binding to the AACUGAC sequence in miR-223. In vitro silencing of STIM1 can promote the expression of miR-223 and inactivate NLRP3 and inflammasome, inhibit the oxidative stress and inflammatory response induced by IAV, reverse cell viability, inhibit cell apoptosis, and thereby reduce the inflammatory damage of lung epithelial cells induced by IAV [60]. MiR-29a binds to the 3′-UTR binding site of the Wnt/Ca signal receptor frizzled5 gene, reduces the expression level of endogenous frizzled5 protein, reduces the mRNA and protein levels of IAV, and also reduces the production of the progeny virus. Moreover, the inhibitory effect of miR-29a on IAV infection was observed in A/PR/8/34, A/WSN/1933, A/OK/3052/09, and A/OK/309/06 H3N2 and other influenza virus strains [61].

### 2.3. Host miRNAs Promote the Replication of IAV

During the interactions between the influenza virus and host, host miRNAs inhibit virus replication directly and indirectly. However, some studies have shown that the miRNAs can negatively regulate the antiviral response pathway, reduce the expression of antiviral factors, and promote the replication of the influenza virus.

Host miRNAs negatively regulate TLR and RLR-mediated innate immune response pathways to promote IAV replication. The expression of miR-21-3p was significantly reduced in A549 cells infected with H5N1, and miR-21-3p down-regulated the expression of basic fibroblast growth factors 2 (FGF2) recombinant proteins, accelerated the replication of H5N1, and inhibited the IFN response. The overexpression of miR-21-3p significantly increased the expression levels of viral genes M1 and NP and viral titers in influenza virus-infected cells and significantly decreased the expression of antiviral factors such as IFN, PKR, MxA, and OAS [62]. In addition, miR-21-3p can promote the replication of IAV by inhibiting the expression of the histone deacetylase-8 (HDAC8)-inhibiting gene by targeting the 3′-UTR gene of HDAC8 [63]. Type I IFN plays an important role in host resistance to influenza virus infection. In type II alveolar epithelial cells infected with IAV, host miR-93 was inhibited in the RIG-I/JNK pathway, and the target protein gene JAK1 of miR-93 was up-regulated. This promoted IFN signal transduction and thus improved antiviral ability. In vivo injection of miR-93 antagonist significantly inhibited IAV infection and protected mice from IAV-related death [64].

Host miRNA negatively regulates NF-κB and IRF-mediated innate immune response signaling pathways to promote IAV replication. As a pro-inflammatory cytokine, zinc finger protein A20 participates in the negative feedback regulation of IL-I/tumor necrosis factor and other types of signal transduction and plays a role in inhibiting the antiviral immune response in the NF-κB and IRF signaling pathways. Influenza virus infection induces the up-regulation of miR-29c expression and stabilizes the expression of A20, thus improving the capacity for viral replication [44]. In IAV-infected monocyte-derived DCs, the regulation level of miR650 was negatively correlated with the mature state of DCs. In IAV-infected monocyte-derived dendritic cells, influenza viruses utilize the host miR-650 to target the 3′-UTR of antiviral factor MxA to reduce the expression of antiviral protein and to improve viral replication [45]. IAV is one of the most common pathogens that cause ARDS. Infected epithelial cells release a large number of inflammatory mediators, mediating endothelial leakage, and neutrophils are recruited into the lung, resulting in decreased epithelial-endothelial barrier function, aggravating alveolar edema, and leading to respiratory failure [65].

These results indicate that some host miRNAs can directly regulate viral gene expression during host-influenza virus interaction. In addition, some host miR-RNAs indirectly regulate the replication of the influenza virus by regulating the antiviral immune response signal in host cells. These findings provide new ideas for a comprehensive understanding of the interaction mechanism between host cells and influenza virus and the discovery of effective targets for antiviral drugs.

**Table 2:** Host miRNAs indirectly regulate influenza A virus replication.

Signaling Pathways ^a^	Target Gene ^b^	miRNA	Cell Type	Regulation Direction ^c^	Virus	Induced Expression ^d^	References
RLRS	RIG-1	miR-485	HEK293T	Promote	H5N1 (A/duck/India/02CA10/2011)	UP	[31]
	RIG-1, IL-6	miR-136	A549	Inhibition	H5N1 (A/duck /Hubei /hangmei01/2006)	UP	[56]
	RIG-1	miR-93	A549	Promote	H1N1 (A/ Puerto Ri-co/8/1934)	DOWN	[64]
TLRS	ARCN1	miR-33a	A549, Hela	Inhibition	H1N1 (A/WSN/33) H9N2 (A/Chicken/Liaoning/1/100)	UP	[66]
	FGF2	miR-194	A549	Inhibition	H1N1 (IAV/ Beijing/501/2009)	UP	[67]
	IRF5	miR-302a	A549	Inhibition	H1N1 (A/FM/1/47)	DOWN	[59]
	IRAK1, MAPK3	miR-7, miR-132, miR-187, miR-1275, miR-200c	A549	Inhibition	H1N1 (A/WSN/33)	UP	[68]
	HDAC1	miR-449b	A549	Inhibition	H1N1 (A/WSN/33)	UP	[69]
	TOLL 4	Let-7e	THP-1	Inhibition	H7N9 (A/Anhui/1/2013)	UP	[42]
NF-κB	IRAK-1, TRAF6	miR-146a	Hela	Inhibition	H1N1 (A/Jing fang/01/1986) H3N2 (A/Lu fang/09/ 1993)	UP	[70]
	TRAF6	miR-144	HBE	Inhibition	H1N1 (A/ Puerto Ri-co/8/34)	UP	[71]
	NFKBIB	miR-4776	MDCK	Inhibition	H1N1 (A/WSN/33)	UP	[72]
	BCL2L2	miR-29c	A549	Inhibition	H1N1 (A/Jing fang/01/1986)	UP	[44]
	NIK	miR-302c	A549	Promote	H3N2 (A/Hong Kong/498/97)	DOWN	[57]
	TRAF6	miR-146a	A549	Promote	H1N1 (A/Jing fang/01/ 1986) H3N2 (A/Lu fang/09/1993)	UP	[70]
	A20	miR-29c	A549	Promote	H1N1 (A/Jingfang/01/1986)	UP	[44]
	HDACB	miR-21-3P	A549	Promote	H5N1 (A/goose/Ji-Lin/ hb/2003) H1N1 (A/Beijing/501/2009)	DOWN	[63]
	FGF2			Promote	H5N1 (A/Hong Kong/156/97)	DOWN	[62]
	IRF3, IRF7	miR-24, gga-miR-30b, miR-142-3	A549	Inhibition	H9N2 (environment/HN/1-18/2007)	UP	[73]
		miR-375, gga-miR-181b	Chicken	Inhibition	H9N2 (environment/HN/1-18/2007)	UP	
	USP3	miR-26a	MDCK	Inhibition	H1N1 (A/WSN/33)	UP	[74]
Jak-STAT	STIM1	miR-223a	A549	Inhibition	H1N1 (A/Puerto Rico/8/34)	UP	[60]
	IFNAR1, STAT2	miR-200a	A549	Promote	H1N1 (r1918 and A/Texas/36/91)	DOWN	[43]
	CUEDC2	miR-324-5p	A549	Inhibition	H5N1 (A/duck/India/02CA10/2011)	DOWN	[39]
	IRF3, IFIT2, MxA	miR-650	NK	Promote	H1N1 (A/Puerto Rico/8/34)	DOWN	[45]
	JNK/c-Jun	miR-206	A549	Inhibition	H1N1 (A/ Puerto Rico/8/34) H3N2 (A/Oklahoma/309/2006)	DOWN	[46]
	STAT3	put-miR-34	HBE	Inhibition	H9N1 (1WF10)	DOWN	[75]
Apoptosis	C0X6C	miR-4276	A549	Inhibition	H1N1 (A/WS/33) H3N2 (A/Aichi/2/68)	UP	[76]
	HMBOX1	ssc-miR-221-3p ssc-miR-222	PAM	Inhibition	H5N1 (A/duck/Anhui/1/2006)	UP	[38]
	MCPIP1	miR-9	A549	Promote	H1N1 (A/PR/8/34) H3N2 (A/Lufang/9/93)	UP	[77]
	IL-6	miR-let-7b-MRE	HBE	Inhibition	H1N1 (A/Nanjing/NJU-108/2009)	UP	[78]
MAPK/ERK	Vimentin	miR-1290	A549	Promote	H1N1 (A/Taiwan/126/2009)	UP	[79]
Muscleblind	Mbnl3	miR-674	Dendritic	Inhibition	H9N2 (A/duck/ Nanjing /01/1999)	UP	[80]
Wnt/β-catenin	frizzled 5 gene	miR-29a	HEK293T A549	Inhibition	H3N2 (A/Oklahoma//309/2006)	UP	[61]
DC/TCell	DR1	miR-203	A549	Inhibition	H5N1 (A/Vietnam/1194/2004	UP	[81]
mTOR	mTOR	miR-101	A549	Inhibition	H5N1 (A/Hatay/2004)	UP	[82]

^a^: Signaling pathways involved in regulation by miRNAs; ^b^: target genes bound by miRNAs in the signaling pathway; ^c^: miRNA mediated regulation of influenza virus replication; ^d^: induced expression of miRNA after influenza virus infection.

## 3. Host LncRNAs Regulates IAV Replication

According to reports, lncRNA, as a new type of regulatory factor, can be induced by the influenza virus and expressed in the cytoplasm or nucleus [83,84]. These lncRNAs can interact with a variety of biological macromolecules, directly or indirectly playing an important role in the host antiviral pathway or influenza virus replication. (See Table 3 for relevant information).

### 3.1. Host LncRNAs Directly Act on Viral Genes to Regulates Influenza Virus Replication

It was found that the host lncRNA could directly target the virus gene and affect virus replication during the process of infection. IAV infection significantly induces two host lncRNAs (PAAN and IPAN) not involved in IFN regulation. LncRNA PAAN enhances the activity of viral RNA polymerase by promoting the assembly of the RNA polymerase complex of the influenza virus, and as a forward regulator of influenza virus replication, ensures the efficient synthesis of virus RNA. LncRNA PAAN synthesis is synchronized with IAV replication [85]. It has been demonstrated that the lncRNA IPAN gene can be hijacked by the IAV in the process of viral infection to assist IAV replication. By stably binding with the viral PB1 protein, the IPAN/PB1 complex forms, preventing the degradation of PB1 and facilitating effective IAV transcription and replication [86]. Lnc45 is a broad-spectrum antiviral factor. Infection with multiple subtypes of influenza viruses such as H5N1, H7N9, and H1N1 can significantly induce up-regulation of Lnc45 expression, and overexpression of Lnc45 can significantly inhibit replication of H1N1, H5N1, and H7N9 viruses. Lnc45 translocates to the cytoplasm from the nucleus during H5N1 virus infection and Lnc45 inhibits polymerase activity and nuclear accumulation NP and PA through its stem ring arms thereby impairs IAV replication [87]. 

### 3.2. Host LncRNAs Indirectly Inhibit IAV Replication by Regulating Intracellular Signals

Host lncRNAs inhibit IAV replication by regulating innate immune signaling pathways mediated by TLRs. The innate immune response mediated by TLRs includes several key factors, such as the expression and activation of PRRs, ISG expression, and IFN production [88]. LncRNA-155 promotes the innate immune response to viral infection by negatively regulating the expression of protein tyrosine phosphatase 1B and mediating the high expression of IFN-β and some key ISGs [89]. Silencing of IFN-stimulated lncRNA-ISR in A549 cells resulted in a significant increase in IAV replication, while the overexpression of ISRs decreased viral replication. IFN-β treatment can induce the expression of ISRs. In hosts without type I IFN receptors, the expression of ISRs induced by viral infection is not significant, and ISRs are regulated by IFN-β during the process of IAV infection. IFN-β also has the ability to inhibit virus replication [90].

Host lncRNA inhibit the replication of IAV by regulating the innate immune response mediated by RLRs. The RLR-mediated innate immune response is an important pathway for lncRNAs to participate in the regulation and inhibition of IAV replication. When the virus invades, the intracellular “whistle” RIG-I recognizes the double-strand RNAs (dsRNAs), transmits antiviral signals through downstream mitochondrial antiviral-signaling (MAVS) proteins, and finally induces the expression of IFNs and proinflammatory cytokines. At the early stage of viral infection, lncRNA z3h7a in the cytoplasm binds to tripartite motif-containing protein 25 (TRIM25) and activates RIG-I. As a molecular scaffold, lncRNA z3h7a stabilizes the TRIM25/RIG-I interaction and enhances TRIM25-mediated K63 ubiquitylation of RIG-I. Thus, the downstream signaling of RIG-I and the antiviral innate immune response are promoted [91]. LncRNA-AVAN enhances k63 ubiquitination of RIG-I by binding with TRIM25. At the same time, lncRNA-AVAN is located 419 bp upstream of forkhead box O3 (FOXO3a); it can improve the expression of FOXO3a through chromatin remodeling, promote neutrophil chemotaxis and recruitment, upregulate the expression of IL-8, and then activate antiviral immunity [92]. The novel lncRNA IVRPIE is a key modulator of the host antiviral response. IVRPIE participates in antiviral innate immunosuppressive IAV replication by promoting IFNs and ISGs. In addition, hnPNP-U interacts with IVRPIE to regulate IFN β1 and ISG transcription by affecting the histone modifications of IRF1, IFN-induced protein with interferon-induced protein with tetratricopetide repeats 1 (IFIT1), IFIT3, Mx1, ISG15, and IFN-induced protein 44-like (IFI44L) [93]. RDUR is a multi-function lncRNA, on the one hand, it enhances host antiviral immunity by positively activating the IRF3 and upregulating ILF2/ILF3, thereby positively regulating the expression of IFNs and key ISGs. On the other hand, experiments demonstrate that deletion of RDUR promotes viral infection through downregulating some crucial antiviral genes but activating the NF-κB-dependent inflammatory response, suggesting that virus-induced expression of RDUR may prevent the host from serious inflammation reaction possibly through a mechanism involving a negative feedback control of NF-κB activation and inflammation [94]. LncRNA EGOT, a long non-coding RNA induced by viral infection, can reduce viral replication by promoting the expression of IFNs through pathways such as PI3K/AKT, MAPKs, and NF-κB. LncRNA EGOT also can affect cell autophagy by regulating expression of ATG7, ATG16L1, LC3II, and LC3 [95,96]. In the process of influenza virus infection, lnc-Cxcl2 could selectively inhibit the expression of Cxcl2 in mouse lung epithelial cells, but not in macrophages, which can attenuate inflammatory damage through feedback inhibition of lung epithelial cells chemokine expression and neutrophil recruitment [97].

### 3.3. Host LncRNAs Indirectly Promote IAV Replication by Regulating Intracellular Signaling

Influenza virus negatively regulates the antiviral immune response mediated by TLRs by inducing the expression of intracellular lncRNA [98]. A/WSN/1933, A/Oklahoma/3052/09, and type I IFNs can induce the significant expression of lncRNA PSMB8-AS1, inhibit the expression of lncRNA PSMB8-AS1, and effectively reduce the expression of IAV genes and the release of progeny IAV virions [99]. As an IFN-inducible gene, lncRNA-MxA is significantly induced and up-regulated during IAV infection. It negatively regulates the transcription of IFN-β by forming a triplet with the IFN-β promoter and plays an important role in the negative feedback loop involved in maintaining immune homeostasis as a negative regulator of the antiviral immune response [100].

RLRs signaling pathway plays a key role in the escape process of the influenza virus. After the influenza virus invades the body, viral proteins will degrade or inactivate RIG-I, MDA5, MAVS, and other monitoring proteins, thus avoiding the immune mechanism of the body [101]. The lncRNA NRAV and lncRNA VIN have been shown to increase viral replication. The expression level of NRAV was significantly decreased in cells infected with IAV. Overexpression of NRAV significantly promoted the replication and virulence of IAV, and knockout of NRAV decreased the replication and virulence of IAV. By changing the histone modification levels of IFITM3 and MxA promoters in key ISGs, NRAV activated H3K4me3 and inhibited H3K27me3, thus negatively regulating the gene transcription of IFITM3 and MxA. Through RNA pull-down and RNA immunoprecipitation (RIP) tests, it was also found that NRAV specifically interacted with the transcription factor zonula occludens-1 nucleic acid-binding protein (ZONAB) to affect the transcriptional regulation of MxA [102]. VIN is a virus-induced lncRNA. Low expression of VIN can inhibit viral replication and significantly reduce viral gene expression. Nuclear localization suggests that VIN and PSMB8-AS1 play a role in the transcription and replication of IAV RNA genomes [103]. lncRNA-lsm3b is an IFN-induced lncRNA. In the late stage of mouse macrophage infection, lnc-lsm3b acts as bait for RIG-I to compete with viral RNA for RIG-I monomer binding, restricting the conformational change in RIG-I protein and preventing further activation of RIG-I. Lnc-lsm3b also reduces IFN-I production to maintain immune homeostasis, and its overexpression in L929 cells interferes with TRIM25-mediated K63 junction ubiquitination of RIG-I during viral infection [104].

A new study found that the lncRNA ACOD1 promotes IAV replication by regulating cell metabolism [105]. LncRNA ACOD1 can be induced by a variety of viruses including IAV. It can directly combine with the metabolic enzyme glutamate-oxaloacetate transaminase (GOT2) in the cytoplasm, improve the catalytic activity of the enzyme, enhance the production of key metabolites required for viral replication, and promote the replication of the influenza virus in A549 cells [106].

The above studies showed that in addition to host lncRNAs participating in the innate immune response pathway to regulate the replication of IAV, influenza viruses can also use host-encoded lncRNAs to negatively regulate the innate immune response of the host.

## 4. Other Host Non-Coding RNAs Regulate the Replication of Influenza A Virus

Vault RNA (vtRNA) is a class of non-coding RNAs in the Vault ribosome complex, which plays a key role in the process of influenza virus infection and replication [107]. PKR is an important part of host innate immunity against viral infection, and the H1N1 influenza virus can induce high expression of vtRNAs in host cells. Silencing of vtRNAs in A549 cells significantly inhibited IAV replication, while overexpression of vtRNAs significantly promoted viral replication. Further studies showed that vtRNAs promote viral replication by inhibiting PKR activation and the subsequent antiviral IFN response. Viral NS1 protein was shown to be an inducer that triggered the upregulation of vtRNAs. In addition, the effective inhibition of PKR by NS1 during IAV infection requires increased expression of vtRNAs [108] (See Table 3 for relevant information). These studies demonstrated that vtRNAs, as host factors utilized by viruses, play an important role in influenza virus antagonism against host innate immunity. However, the molecular mechanism by which NS1 regulates the expression of vtRNAs remains to be studied.

Virus–host interactions are complicated processes, and multiple cellular proteins promote or inhibit viral replication through different mechanisms. CircRNAs are a newly discovered class of endogenous regulatory RNAs that are widely expressed and characterized by a covalent closed-loop structure without a 5′ cap or 3′ tail. In recent years, a growing number of circRNAs have been reported to play important roles in a variety of human diseases, including in dynamic interactions between the virus and host [109,110,111,112,113]. H1N1 infection induced the overexpression of circ-GATAD2A. With circ-GATAD2A knockout in the A549 cell line, autophagy was enhanced while H1N1 replication was suppressed. In contrast, overexpression of circ-GATAD2A in the A549 cell line suppressed autophagy and promoted H1N1 replication. Further research showed that circ-GATAD2A interacted with vacuolar protein sorting 34 (VPS34) and the inhibition of autophagy promoted the replication of H1N1 [114]. The researchers found and identified a novel intron circRNA named AIVR that was upregulated in A549 cells infected by the influenza virus. Silencing AIVR significantly promoted the replication of the influenza virus in A549 cells, as a miRNA sponge, AIVR is mainly located in the cytoplasm. It plays an antiviral role by absorbing miRNA and promoting the expression of CREBBP so as to promote the expression of IFN-β [113].

**Table 3:** Regulation of influenza A virus replication by host lncRNAs.

LncRNA		Target Gene ^a^	Cell Type	Regulation Direction ^b^	Virus	Induced Expression ^c^	References
LncRNA IPAN		PB1	A549	Promote	H1N1 (A/WSN/33)	UP	[86]
LncRNA PAAN		PA	A549	Promote	H1N1 (A/PR/8/34)	UP	[85]
Signaling pathways ^d^	LncRNA	Target gene ^e^	Cell type	Regulation direction ^b^	Virus	Induced expression ^c^	References
RLRs	LncRNA NRAV	ZONAB	A549	Promote	H1N1 (A/WSN/33)	DOWN	[115]
	LncRNA Lsm3b	RIG-I	L929	Promote	H1N1 (A/ Puerto Rico /8/1934)	UP	[104]
	LncRNA VIN	Nuclear	A549	Promote	H1N1 (A/WSN/33)	UP	[103]
	LncRNA-155	PTP1B	A549	Inhibition	H1N1 (A/Puerto Ri-co/8/1934)	UP	[89]
	LncRNA IVPRIE	RIG-I	A549	Inhibition	H1N1 (A/Puerto Ri-co/8/1934)	UP	[93]
	LncRNA NEAT1	SFPQ	Hela	Inhibition	H1N1 (A/WSN/33)	UP	[116]
	LncRNA ISG20	miR-326	A549 HEK293T	Inhibition	H1N1 (A/Puerto Ri-co/8/34)	UP	[117]
	LncRNA ISR	IFN-β	A549	Inhibition	H1N1 (A/California/04/2009)	UP	[90]
	LncRNA AVAN	TRIM25	A549	Inhibition	H7N9 (A/Hebei/01/2013)	UP	[92]
	lncRNA IFITM4P	miR-24-3p	A549	Inhibition	H1N1 (A/Shanghai-Jiading/SWL1970/2015)	UP	[118]
TLRs	LncRNA TSPOAP1	NF-κB	A549	Promote	H1N1 (A/PR/8/34) H3N2 (A/Lufang/9/93)	UP	[119]
	LncRNA-MxA	IFN-β	A549	Promote	H1N1 (A/WSN/33)	UP	[100]
	PSMB8-AS1	IFN	A549	Promote	H1N1 (A/Puerto Ri-co/8/34)	UP	[99]
Cell metabolism	LncRNA-ACOD1	GOT2	A549	Promote	H1N1 (A/WSN/33)	UP	[106]
NF-κB	LncRNA TUG1	miR-145-5p	DHBE	Inhibition	H3N2	UP	[120]
	LncRNA RDUR	RIG-I/MAVS /NF-κB	A549	Inhibition	H1N1 (A/Shanghai-Jiad-ing/SWL1970/2015)	UP	[94]
Other ncRNAs in the host regulate influenza A virus replication							
Signaling pathways ^d^	NcRNA	Target gene ^e^	Cell type	Regulation direction ^b^	Virus	Induced expression ^c^	References
Post transcriptional regulation	Circ_0050463	microRNA-33b-5p	A549	Promote	H1N1 (A/Lufang/9/93)	UP	[121]
Autophagy regulation	Circ-GATAD2A	VPS34	A549	Promote	H1N1 (A/ Puerto Ri-co /8/34)	UP	[114]
PKR	vtRNAs	NS1	A549	Promote	H1N1 (A/WSN/33)	UP	[108]

^a^: LncRNAs directly target key genes of viruses; ^b^: ncRNA mediated regulation of influenza virus replication; ^c^: induced expression of ncRNA after influenza virus infection; ^d^: signaling pathways involved in regulation by ncRNA; ^e^: target genes bound by ncRNAs.

## 5. Host Non-Coding RNAs Regulate the Replication of Other Influenza Virus

Influenza B viruses have a limited host range compared to influenza A viruses, the mutation rate was low, and is not easy to cause a pandemic. In the past few decades, there have been many studies on the interaction between host and influenza B virus Host noncoding RNA plays an important role in directly or indirectly regulating the replication of influenza B virus. (See Table 4 for relevant information).

According to reports, the hsa-miR-30e-3p is one of the miRNAs regulating influenza B virus infection, it is able to directly inhibit the expression of NA, NP genes of the influenza B virus (influenza B virus B/Thailand/CU-B5522/2011 representing the Victoria lineage) [122]. Replication and transport of Zika virus (ZIKV) and porcine reproductive and respiratory syndrome virus (PRRSV) in host cells require karyopherin alpha 6 (kpna6). In MDCK cells infected with influenza B virus (Victoria lineage (B/Thailand/CU-B5522/2011) or Yamagata lineage (B/Massachusetts/2/2012)), five miRNAs, including cfa-miR-197, cfa-miR-215, cfa-miR-361, cfa-miR-1841 and cfa-miR-1842, were significantly upregulated. The cfa-miR-197 mediates KPNA6 silencing by specifically binding to the 3′-UTR (GUGGUGA/UGGUGAA) of KPNA6. Therefore, this miRNA can inhibit the replication of the influenza B virus [123]. In chicken embryos infected with the influenza B virus, the expression level of miRNA induced by viral infection in the spleen was higher than in lungs, including miR-34c, -34b, -1b, -1a, -206, and -499. These miRNAs are also induced by infection of avian influenza virus, and most of them are involved in regulating immune responses (miR-34c, -34b, -1b, -1a and -206). Studies have shown that gga-miR-30d inhibited the replication of IBV by targeting ubiquitin-specific peptidase 47 (USP47) in HD11 cells. However, also, in HD11 cells miR-146a-5p promoted IBV replication by targeting interleukin 1 receptor-associated kinase 2 (IRAK2) and TNF receptor superfamily member 18 (TNFRSF18) [123,124]. On this basis, the researchers further analyzed the changes of lncRNAs in IBV-infected HD11 cells and mapped the competitive endogenous RNA (ceRNA) regulation network of lncRNA-microRNA-mRNA, and the researchers also found that lncRNA MSTRG.21445.2 may regulate IBV infection by competing for gga-miR-30d and miR-146a-5p for mRNA with USP47, IRAK2, and TNFRSF18. These results provide new insights into the relationship between host non-coding RNA and replication of IBV, but most of the results are just based on bioinformatics analysis. Therefore, these findings should be further confirmed by laboratory studies [125].

Influenza C virus is a common pathogen of acute respiratory diseases, and Children are susceptible to the influenza C virus. Influenza D is a newly discovered virus in cattle and pigs, which can cause mild to moderate respiratory diseases and target the upper and lower respiratory tract. IDV and ICV have about 50% homology. The current studies on these two influenza viruses focus on the interaction between virus and host proteins. For example, the NS1 of ICV and IDV, similar to the NS1 of IBV and IAV, can be overexpressed in the infected host cells and inhibit the IFN function of the host cells; ANP32A-mediated influenza virus replicase assembly [126]; TMPRSS2 protein cleaves the HE of ICV and activates ICV [127]. However, there are few reports on the regulation of type C and type D influenza viruses by host non-coding RNA. It is believed that there will be more new research progress in this direction with in-depth research and understanding of the infection mechanism of type C and type D influenza viruses in the future.

**Table 4:** Regulation of miRNA on replication of influenza viruses other than influenza A virus.

miRNA	Target Gene ^a^	Cell Type	Regulation Direction ^b^	Virus	Induced Expression ^c^	Binding Site of Target ^d^	References
hsa-miR-30e-3p	NA, NP	A549	Inhibition	IBV (Influ-enza B virus B/Thailand/CU-B5522/2011 representing the Vic-toria lineage)	UP	GAUGUCU GAACUGAAA	[122]
Signaling pathways ^e^	miRNA	Target gene ^f^	Cell type	Regulation direction ^b^	Virus	Induced expression ^c^	References
TLRs	cfa-miR-197	KPNA6	MDCK	Inhibition	IBV Victoria lineage (B/Thailand/CU-B5522/2011), or Yamagata lineage (B/Massa Chu-setts/2/2012)	UP	[128]
	gga-miR-30d	USP47	HD11	Inhibition	IBV (Beaudette strain)	DOWM	[124]
	gga-miR-146a-5p	IRAK2, TNFRSF18	chickens	promote	IBV (Beaudette strain)	UP	[123]

^a^: ncRNAs target key genes; ^b^: ncRNA mediated regulation of influenza virus replication; ^c^: induced expression of ncRNA after influenza virus infection; ^d^: sequences of miRNA binding site of target (5′-3′); ^e^: signaling pathways involved in regulation by ncRNA; ^f^: target genes bound by ncRNAs.

## 6. Discussion

In recent years, non-coding RNAs have attracted extensive attention from researchers. In this paper, through a comprehensive analysis of relevant studies on the regulation of host non-coding RNAs involved in the replication of IAV, we found that host noncoding RNAs mainly regulate influenza virus replication directly and indirectly. Some of them play a role in inhibiting the replication and proliferation of the virus, while others assist the virus to escape the monitoring system of the host immune response and invade the host cells faster by interacting with the virus genes. This provides a new strategy for the development of anti-influenza drugs targeting key genes of the virus.

In addition to directly regulating the expression of influenza virus genes, host non-coding RNA can also participate in the coordination of intracellular signaling and the expression of antiviral factors, and indirectly regulate the replication of the influenza virus. With further research on non-coding RNAs, how these host non-coding RNAs regulate the expression of antiviral factors in the process of innate immune response and play an indirect role in the regulation of the replication and proliferation of influenza viruses will become clearer, and these non-coding RNAs will hopefully become new candidate drugs for anti-influenza virus research.

**Figure 2:**
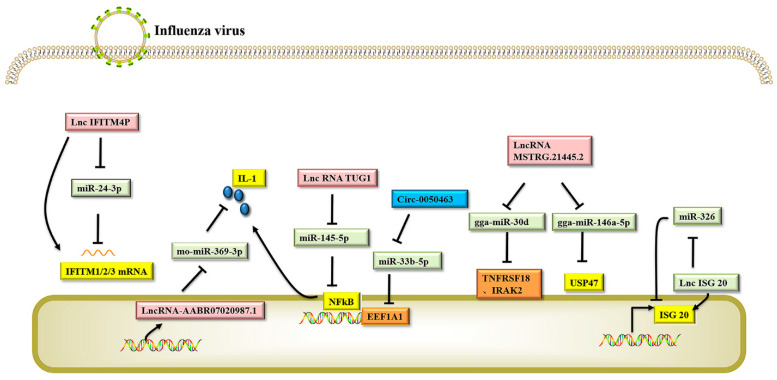
Competitive endogenous RNAs involved in regulating influenza virus replication. The ceRNA interaction network has more complex regulatory mechanisms compared to the simple host non-coding RNA regulating influenza virus replication. The regulatory process involves mutual regulation between non-coding RNA, and also non-coding RNA interaction with host proteins, ultimately enabling regulation of influenza virus replication through a series of complex regulatory mechanisms. (The Pink/blue boxes indicate lncRNA/circRNA that can bind to microRNA; green box indicates the microRNA that binds to the key antiviral protein of the host; The yellow/orange box indicates the host protein that can inhibit/promote the expression of influenza virus during antiviral process).

CeRNAs provide a new regulation mode for gene expression. Studies have shown that compared to simple miRNA-mRNA regulation, the ceRNA regulation network is more complex and generally consists of an integrated regulation network involving circRNA-miRNA-mRNA and lncRNA-miRNA-mRNA. Currently, studies on ceRNAs are mainly focused on their role in the regulation of tumor genesis, and there are few reports on how ceRNAs regulate influenza virus replication. LncRNAs and circRNAs have abundant miRNA binding sites, so they can bind the MERS of miRNAs, like a sponge, to form ceRNAs, prevent miRNAs from binding with target gene mRNAs, and regulate the expression of the target genes (see Figure 2 for relevant information) [129]. A new study found that as a ceRNA, the lncRNA IFITM4P can be induced and expressed by multiple H1N1 strains and other pathogenic viruses. In vitro and in vivo experiments demonstrated that IFITM4P can participate in the natural immune antiviral response through the lncRNA IFITM4P-miR-24-3p-IFITM1/2/3 regulatory network and is a potential antiviral host factor [118]. By analyzing the expression profile of lncRNAs before and after influenza virus infection, researchers found that the expression of lnc-ISG20 in infected cells was significantly up-regulated. IAV infection triggers the transcription of type I interferons, such as IFN-β. IFN-β stimulates the transcription of downstream genes (ISG20 and lnc-ISG20). miR-326 inhibited the translation of ISG20 by targeting the 3′UTR of ISG20 mRNA, thus promoting the replication of the virus. Lnc-ISG20 acts as a ceRNA by reducing miR-326 binding to target ISG20 mRNA and enhances the translation of ISG20, which in turn inhibits IAV replication. [117]. The NF-κB pathway is positively regulated by the lncRNA TUG1 and negatively regulated by miR-145-5p. Influenza virus can induce airway hypersensitivity in chronic obstructive pulmonary disease (COPD). TUG1 positively regulates airway inflammation mediated by the NF-κB pathway by binding miR-145-5p. Inhibition of TUG1 can inhibit the expression of the NF-κB pathway and its downstream pro-inflammatory cytokines IL-1β and TNF-α, and significantly reduce airway inflammation [120]. LncRNA-AABR07020987.1 positively affected the expression of IL-17A by acting as a ceRNA to compete with IL-17A mRNA for binding sites of mo-miR-369-3p [130]. Similarly, in A549 cells infected with IAV, Circ-0050463 an endogenous miRNA-33b-5p sponge, can isolate and inhibit the activity of miRNA-33b-5p, activate the expression of eukaryotic translation elongation factor 1 alpha 1 (EEF1A1), and promote IAV replication through the mir-33b-5p/EEF1A1 axis [121]. These results indicate that compared with the miRNA regulatory network, the ceRNA regulatory network is more delicate and complex, and involves more RNA molecules, including mRNA, lncRNA, miRNA, and circRNA, which provides a new perspective for researchers to conduct transcriptome studies. This knowledge is also helpful to explain some biological phenomena more comprehensively and deeply.

In the face of cunning and changeable influenza virus, effectively activating the immune response to the conserved domains of the virus is an effective method to realize a broad-spectrum influenza vaccine [131]. Specific antibodies demonstrated in mouse, ferret models targeting viral protein conserved domains, such as matrix protein M2 or HA stem domains, can be effective to treat or prevent infection of different influenza strains [132]. Studies have shown that it is promising to enhance the immune protective effect of vaccines by using host non-coding RNA. For example, murine models have demonstrated that docetaxel can upregulate the Th1, Th2 immune responses, and has an influenza A H1N1 cleavage vaccine adjuvant effect. While the enhanced immune response may be related to the upregulation of miR-155 expression as detected in docetaxel-stimulated RAW264.7 cells [133]. Under direct regulation, miRNA can inhibit virus replication by binding virus mRNA. For example, inserting miR-let-7b into the PB1 gene of the H1N1 influenza virus can reduce the replication and proliferation ability of the virus in cells and animals [78]. In A549 cells, hsa-miR-1-3p decreases the expression of NP genes and suppresses replication of PR8 and H3N2 [134]. While miRNA-192-5p was inserted into the nuclear protein (NP) genomic fragment of the influenza virus to prepare live attenuated vaccines, cells and mice vaccinated with miRNA showed higher survival compared with general vaccine [135]. This research evidence suggests that the modification of key viral segments with host noncoding RNA as a strategy can be used to study and develop live-attenuated influenza vaccines.

In conclusion, host non-coding RNAs affect the process of viral infection by regulating the expression of host or virus-related genes and play an important role in the complex relationship between the host and virus regulation, facilitating a state of balance between the virus and host. The relationship between non-coding RNAs and viral infection still has broad research prospects. However, the mechanism of non-coding RNAs in influenza virus infection remains to be further studied. On the one hand, the current research mainly focuses on the function of non-coding RNAs in influenza virus infection, and it is not clear whether non-coding RNAs play a role in the escape process of influenza virus. On the other hand, influenza virus infection can induce the differential expression of a large number of non-coding RNAs in host cells. Only a few studies have confirmed their function in the interaction between virus and host. The role of many non-coding RNAs in the process of influenza virus infection is still poorly understood. Further clarification of the interaction mechanism will help researchers better understand the precise regulatory mechanism of these host non-coding RNAs during the process of IAV infection. It will also help provide promising targets for the development of antiviral strategies by reducing key regulatory factors associated with viral infection and enhancing innate immune responses.
